# Imaging and Manipulation of Plasma Membrane Fatty Acid Clusters Using TOF-SIMS Combined Optogenetics

**DOI:** 10.3390/cells12010010

**Published:** 2022-12-20

**Authors:** Chi Zhang, Kenji Kikushima, Mizuki Endo, Tomoaki Kahyo, Makoto Horikawa, Takaomi Matsudaira, Tatsuya Tanaka, Yusuke Takanashi, Tomohito Sato, Yutaka Takahashi, Lili Xu, Naoki Takayama, Ariful Islam, Md. Al Mamun, Takeaki Ozawa, Mitsutoshi Setou

**Affiliations:** 1 Department of Cellular and Molecular Anatomy, Hamamatsu University School of Medicine, 1-20-1 Handayama, Higashi-ku, Hamamatsu, Shizuoka 431-3192, Japan; 2 International Mass Imaging Center, Hamamatsu University School of Medicine, 1-20-1 Handayama, Higashi-ku, Hamamatsu, Shizuoka 431-3192, Japan; 3 Department of Chemistry, School of Science, The University of Tokyo, 7-3-1 Hongo, Bunkyo-ku, Tokyo 113-0033, Japan; 4 Hiroshima Research Center for Healthy Aging, Department of Molecular Biotechnology, Graduate School of Advanced Sciences of Matter, Hiroshima University, 1-3-1 Kagamiyama, Higashi-Hiroshima, Hiroshima 739-8530, Japan; 5 Foundation for Promotion of Material Science and Technology of Japan, 1-18-6 Kitami, Setagaya-ku, Tokyo 157-0067, Japan; 6 Department of Systems Molecular Anatomy, Institute for Medical Photonics Research, Preeminent Medical Photonics Education & Research Center, 1-20-1 Handayama, Higashi-ku, Hamamatsu, Shizuoka 431-3192, Japan

**Keywords:** plasma membrane fatty acids, TOF-SIMS, optogenetics, membrane heterogeneity, fatty acid imaging

## Abstract

The plasma membrane (PM) serves multiple functions to support cell activities with its heterogeneous molecular distribution. Fatty acids (FAs) are hydrophobic components of the PM whose saturation and length determine the membrane’s physical properties. The FA distribution contributes to the PM’s lateral heterogeneity. However, the distribution of PM FAs is poorly understood. Here, we proposed the FA cluster hypothesis, which suggested that FAs on the PM exist as clusters. By the optogenetic tool translocating the endoplasmic reticulum (ER), we were able to manipulate the distribution of PM FAs. We used time-of-flight combined secondary ion mass spectrometry (TOF-SIMS) to image PM FAs and discovered that PM FAs were presented and distributed as clusters and are also manipulated as clusters. We also found the existence of multi-FA clusters formed by the colocalization of more than one FA. Our optogenetic tool also decreased the clustering degree of FA clusters and the formation probability of multi-FA clusters. This research opens up new avenues and perspectives to study PM heterogeneity from an FA perspective. This research also suggests a possible treatment for diseases caused by PM lipid aggregation and furnished a convenient tool for therapeutic development.

## 1. Introduction

The plasma membrane (PM) serves several roles as both a barrier and a gateway of communication between the interior and exterior of the cell [1]. The heterogeneity of the PM is the basis of its multiple functions [2]. How to explain and describe this heterogeneity has recently emerged as one of the most intriguing aspects of membrane research. Researchers have characterized the heterogeneity from the perspective of various PM molecule distributions. Based on the fluid mosaic model proposed by Singer and Nicolson in 1972, Kai Simons presented the concept of the “lipid raft” [2,3,4]. The lipid raft model describes the heterogeneity of the PM in terms of cholesterol and sphingolipids [4,5]. Based on this, Akihiro Kusumi proposed a hierarchical three-layer mesoscale domain structure from the standpoint of the membrane skeleton (actin) and membrane compartment to further interpret the PM heterogeneity [5,6,7]. Although lipid raft studies remain controversial and unsolved mysteries [8,9], it has inspired the further understanding of PM heterogeneity, such as the effect of lipids on the physical properties and phase behavior of biological membranes [10].

Fatty acids (FAs) are the acyl chains of phospholipids. As the hydrophobic part, FAs are the major constituent molecule of the PM. The length and saturation of FAs directly affect the physical properties of the membrane [11]. Lipid packing and molecular ordering (distribution) of FAs are properties that strongly influence PM heterogeneity and lateral diffusion coefficient [12]. The FA distribution thus contributes to the lateral heterogeneity of the PM. However, the distribution of PM FAs remains unrevealed.

Mass spectrometry imaging enables two-dimensional detection and identification of lipid molecules on the surface of biological tissues through various ionization methods [13]. The three main ionization methods are matrix-assisted laser desorption/ionization (MALDI) [14], desorption electrospray ionization (DESI) [15], and secondary ion mass spectrometry (SIMS) [16], each with its own strengths and weaknesses. SIMS uses high-energy ion bombardment of the tissue surface to obtain molecular information about the sample. Time-of-flight combined SIMS (TOF-SIMS) has a spatial resolution of less than 50 nm and is currently the only technique capable of imaging lipids at the single-cell and subcellular levels [16,17,18]. We previously developed the TOF-SIMS method to image cellular FAs at the single-cell level [19].

Molecular cell biology research has also evolved massively with the development of various techniques. Distinct from conventional pharmacological means, a novel method called optogenetics has been developed [20]. It is based on protein conformational changes upon illumination [21]. Optogenetics possesses a higher specificity and spatiotemporal speed for molecule manipulation [22]. This optical manipulation has been applied for organelle movement such as peroxisomes, lysosomes and mitochondria [23]. Furthermore, optogenetics has been applied to manipulate the metabolism of phosphatidylinositide by controlling the activity of the phosphatases [24]. These studies suggest a potential application of optogenetics to lipid research.

We proposed the FA cluster hypothesis in this study to characterize the PM FA distribution. This hypothesis suggests that PM FAs exist as clusters. We verified the FA cluster hypothesis from both imaging and manipulation perspectives. We imaged PM FAs by TOF-SIMS and observed clustered distribution of FAs. By translocating the endoplasmic reticulum (ER), the center of lipid biosynthesis, we could manipulate and change the distribution of PM FAs using optogenetic tools. Using TOF-SIMS, we imaged and compared the distribution of PM FAs before and after manipulation. Our findings revealed that PM FAs were present as clusters both with and without manipulation. The optical manipulation reduced the clustering degree of the PM FAs by transforming them into a relatively homogeneous distribution. Furthermore, we discovered that the distribution of multi-FA clusters containing more than one FA had changed upon the manipulation. The optical manipulation also decreased the probability of multi-FA cluster formation.

In this study, we describe the heterogeneity of PM from the standpoint of FAs, which provides a new perspective to explain the formation of PM heterogeneity. In addition, we present a robust and convenient tool for studying PM FAs.

## 2. Materials and Methods

### 2.1. Plasmids and Reagents

We created a pair of constructs to manipulate the ER morphology: Venus-iLID-cytochrome b5, Kif1a-tagRFP-SspB. The Kif1a were amplified from plasmid: Kif1a-TOM-ePDZ which was a kind present from Dr. Kapitein and Dr. Petra (Utrecht University, 3584 CH Utrecht, The Netherlands) [25]. The cDNAs of Venus-iLID and tagRFP-SspB were amplified from pLL7.0: Venus-iLID-CAAX (from KRas4B) (Addgene, Watertown, MA, USA) and pLL7.0: tgRFPt-SSPB WT (Addgene, Watertown, MA, USA), respectively. The cDNA of cytochrome b5 was generated by PCR [26].

The reconstruction of plasmids was performed inside a competent cell called ‘stellar cells’ according to a protocol ‘In-fusion’(TAKARA). For plasmids construction confirmation, we applied the FastGene mini-kit (Nippon Genetics, Tokyo, Japan) for rapid DNA extraction.

We prepared the plasmids for transfection by Xtra-midi (Macherey-Nagel, Allentown, PA, USA) with a large-volume high-purity extraction and purification.

### 2.2. Cell Culture, Transfection, and Illumination

We applied CV-1 in Origin Simian-7 (COS-7) cells for our experiments (Japanese Collection of Research Bioresources Cell Bank, Ibaraki, Japan). The cells were grown in Dulbecco’s modified Eagle medium (DMEM) (Gibco Thermofisher Scientific, Tokyo, Japan) containing 10% fetal bovine serum (FBS) in a humidified environment at 37 °C, 5% CO_2_.

For transient transfection, we used Lipofectamine 2000 following the protocol from the manufacturer.

We used a light-emitting diode (LED) panel (TH2-211X200BL, CCS Inc., Kyoto, Japan) for the illuminated culturing. The LED panel was supported by a power source (PD3-10024-8-PI, CCS Inc., Kyoto, Japan) connected to an auto-switch-off timer (FT-022, Tokyo Garasu Kikai Co., Ltd., Tokyo, Japan). The panel was set at 24V LIGHT channel with brightness set at 24 which would stably generate blue light with a peak of 465 nm. The cells were displayed on the panel and the power kept on for continuous illumination.

### 2.3. Live Imaging

The cells were maintained in 3.5 cm glass-bottom dishes (IWAKI, Tokyo, Japan), and the media was exchanged into DMEM without phenol red (Gibco) before observation. Cells were placed in a temperature chamber (TOKAI HIT, Fujinomiya, Japan) for live imaging. This temperature chamber kept the cells in a 37 °C, 5% CO_2_ environment.

The observation was carried out using a Leica TCS SP8 system (Leica, Tokyo, Japan) with a Leica DMi8 microscope equipped with a 63/NA-1.40 liquid immersion objective and Type F Immersion Liquid (Leica). The confocal microscope was controlled using the LAS X software (Leica, Tokyo, Japan). The Venus-fused protein was imaged and the iLID-SspB dimerization was activated using laser light (488 nm). RFP-fusion protein imaging was carried out using laser light (552 nm).

### 2.4. TOF-SIMS Sample Preparation

For TOF-SIMS, cells were seeded on indium tin oxide (ITO) glass. For sterilization, the ITO glasses were immersed in 100% ethanol overnight. The ITO cover glasses were then coated in a 35 mm dish with 1 mL of 5 g/mL fibronectin in phosphate-buffered saline (PBS) and incubated at 37 °C for 1 h. The fibronectin solution and blocked glasses/dishes were then removed and incubated at 37 °C for 1 h with 1 mL of 1% bovine serum albumin (BSA) in PBS. Finally, cells were seeded onto ITO cover glasses in a 35 mm dish after washing the dishes/glasses twice with PBS and once with DMEM (+).

Before TOF-SIMS analysis, the cells were fixed. We used a glutaraldehyde and uranyl acetate mixture to fix the cells. To make 1 mL of the mixture, we mixed 125 μL of 20% glutaraldehyde in water, 250 μL of 4% uranyl acetate in water, 200 μL of 0.5 M sucrose in water, and 425 μL water. To remove precipitation, the mixture was incubated in a water bath at 37 °C for 10 min before being centrifuged at 1500 rpm for 1 min. The cells were washed twice with 1 mL of 150 mM ammonium acetate (pH 7.4) in a 35 mm dish before being treated with 1 mL mixture. The cells were incubated with the mixture for 15 min at 37 °C then washed twice with 150 mM ammonium acetate [19].

### 2.5. TOF-SIMS

Measurements were performed using an ION TOF TOF-SIMS V-200 instrument. The instrument was equipped with a Bi liquid metal ion gun. To prevent the surface of the sample from being charged we did not apply the gas cluster ion beam during the analysis. We first took surface spectra from an area of 500 μm^2^ in order to identify the species present at the respective surfaces. Here, the spectrometry mode was used (mass resolution: m/Δm: 6000–8000; the focus of the ion beam: 3–4 μm). The Bi^5+^ primary ions which would not destroy the molecular structure were applied with a primary acceleration voltage of 30 kV.

Following this, mass-resolved secondary ion images were acquired using Bi^3+^ in fast imaging mode and assisted with delayed extraction mode (mass resolution: 2000–2500; the focus of the ion beam: 200–300 nm). 30 kV was the primary acceleration voltage. The primary ion dose for all analyzed samples ranged from 9.3 × 10^11^ to 2.1 × 10^12^ (ions/cm^2^), resulting in minimal surface damage. The fields of view ranged from 10 to 500 μm^2^. Data were collected from several image fields on each of the six sections that were analyzed.

### 2.6. Lipid Extraction

To extract the whole lipids from the cells, we applied a modified Bligh–Dyer method which was performed as follows [27]. First, for a 6 cm dish, the cells were washed with 1 mL PBS twice and 3 mL of PBS added into the dish. The cells were then collected with a cell-scrapper and moved to 1.5 mL glass centrifuge tubes (IWAKI, Tokyo, Japan). We used centrifugation at 1200 rpm for 5 min to remove the PBS and subsequently added 0.8 mL PBS to the tubes. After collecting the cells in the PBS, we then added 1 mL chloroform and 1 mL methanol (FUJIFILM Wako Pure Chemical, Osaka, Japan) with glass tips (CORNING, Tokyo, Japan) and vortexed the mixture. Then, we added 1 mL chloroform and vortexed again. Finally, we added 1 mL acetic acid (Wako) and vortexed once again. After vortex, the mixtures were centrifuged at 1000 rpm for 15 min. After centrifugation, the organic layer (bottom layer) was extracted and dried using a SpinDryerStandard VC-96R connected to a VA-500R (TAITEC, Koshigaya, Japan) and kept at −80 °C until determination.

### 2.7. LC-MS/MS

The lipids were dissolved in methanol and transferred into a glass vial (Waters) specified for LC-MS with a glass insert (Systech, Yokohama, Japan) in it; a specific safe cap (Thermo Scientific, Tokyo, Japan) was screwed onto it for safe moving. Cellular lipids were retrieved and processed by a Q Exactive™ Hybrid Quadrupole-Orbitrap™ mass spectrometer attached to an Ultimate 3000 system (Thermo Scientific) for analysis. A 10 µL lipid sample was infused into the machine and isolated in an Acculaim 120 C18 column (150 mm × 2.1 mm, 3 µm) (Thermo Scientific). The composition of the flow phase was depicted separately below by setting up the mobile phase as two parts, A and B. Part A consisted of water–acetonitrile–methanol (2:1:1 *v/v/v*), 5 mM ammonium formate, and 0.1% formic acid. The flow phase B part consisted of acetonitrile–isopropanol (1:9 *v/v*), 5 mM ammonium formate, and 0.1% formic acid. The elution flow rate was programmed at 300 µL/min using a series of linear gradients beginning with 20% solvent B, increasing linearly to 100% B within 50 min, sustaining 100% B to 60 min, then reducing linearly to 20% B between 60 min and 60.1 min, and terminating with 20% B within the final 10 min. The total operation time was 70 min and conditions of the MS instrument performed were as below: probe heater temperature, 350 °C; S-lens RF level, 50; capillary temperature, 250 °C; auxiliary flow, 15; sheath gas flow, 50; sweep gas flow, 0; spray voltage in positive mode, 3.5 kV, and negative mode, 2.5 kV. Quantitative conditions in full mass spectrometry modality were the following: AGC target, 1 × 10^6^; mass resolving power, 70,000 (FWHM); recorded *m/z* range, 220–2000; maximum sample time, 100 ms.

### 2.8. Image Analysis and Data Analysis

**Figure 1:**
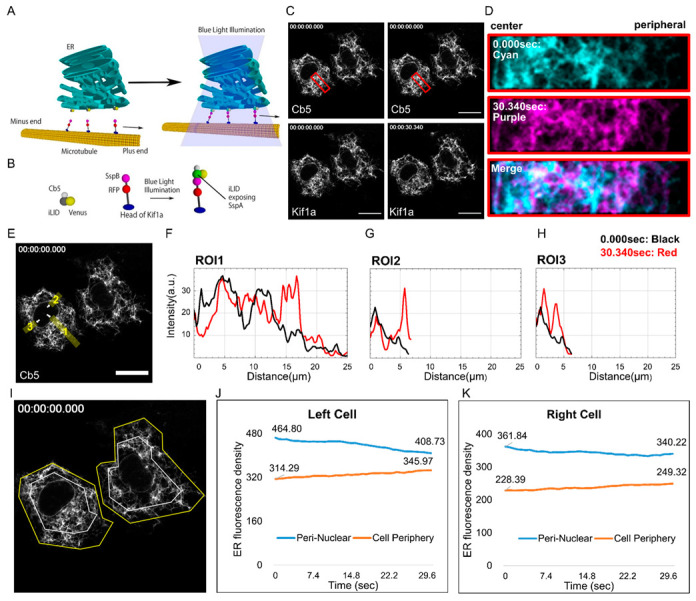
Design and realization of the optogenetic tool. (**A**) The illustration of the optogenetic tool. (**B**) The two plasmids designed for constituting the tool: Venus-iLID-SspB and Kif1a-tagRFP-SspB. (**C**) The time-lapse images of COS-7 cells introduced with the optogenetic tools. Fluorescence signals representing Kif1a and Cb5 are shown. The start point was defined as 0.000 s, the moment the blue light turned on. The evaluation point was set after 30.340 s illumination. (**D**) Red frames in (**C**) are aligned and zoomed for comparison by the two colors (0.000 s: Cyan, 30.340 s: Purple). (**E**) ROIs taken in Cb5 fluorescence images for plot profile analysis at different directions (white arrows: the “0” for the horizontal axis of the plot file). (**F**–**H**) The plot profile of ER fluorescence in ROI 1-3. The charts indicate that the endoplasmic reticulum (ER) was translocated centrifugally by the optogenetic tool. (**I**) ROIs with the same area were taken in Cb5 fluorescence images for intensity comparison (perinuclear: white frame; pericellular: the space between white and yellow frames). (**J**,**K**) The line graph shows the Cb5 fluorescence intensity changes in the perinuclear and pericellular area of the left and right cell. Scale bars: 25 µm.

**Figure 2:**
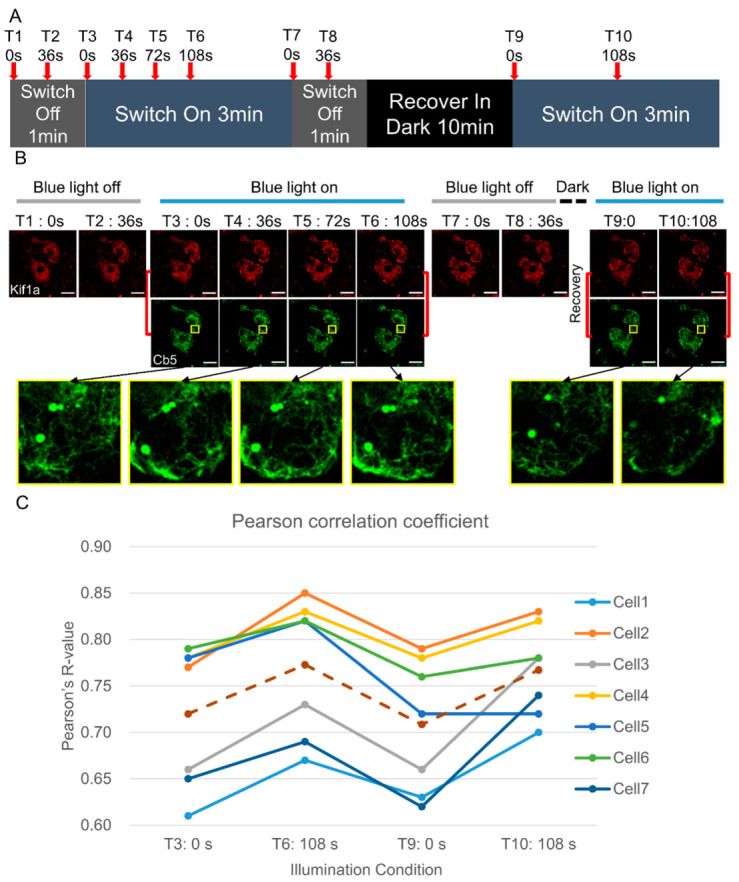
Live-imaging and quantitative analysis showing the reversibility and repeatability of the optoKiC. (**A**) The time window contained two rounds of activation with a 10 min dark interval. Red arrow: the time-point at which images were taken. (**B**) The series images of the live cells introduced with optoKiC were taken at 10 time-points, which showed the ER translocation during different light conditions. Yellow frame: zoomed images for a part of the cell from the center to the periphery. Black arrows: corresponding images and frames. Red lines: Paired images for Pearson’s *R* value calculation. (**C**) The co-localization analysis using the Image J Coloc2 plugin showed the reversibility and reusability of the ER translocation. The Pearson’s *R* value representing the interaction between iLID and SspB, using images of two channels as a group (Image connected by red lines in **B**) Scale bars: 25 µm.

**Figure 3:**
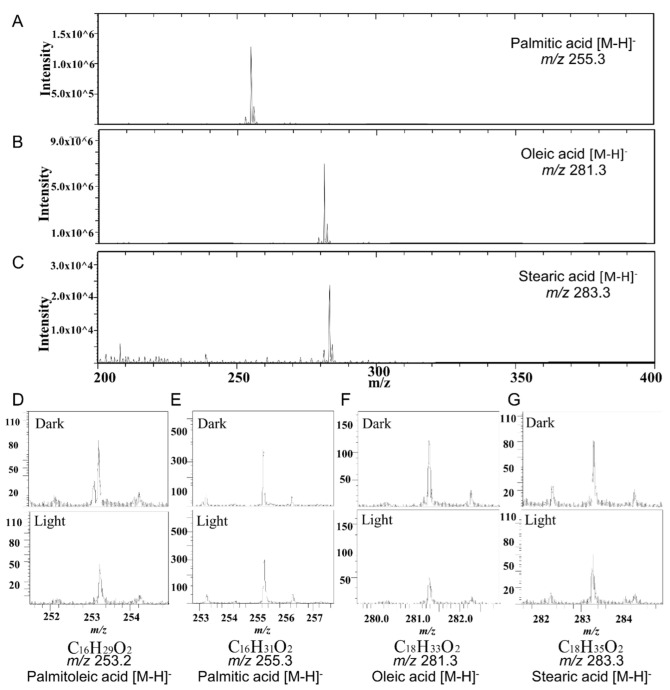
Detection of fatty acids (FAs) with the usage of time-of-flight secondary ion mass spectrometry (TOF-SIMS). (**A**–**C**) Negative mode TOF-SIMS mass spectra of standard palmitic acid (PA), oleic acid (OA), and stearic acid (SA). (**D**–**G**) Negative mode TOF-SIMS mass spectra of palmitoleic acid (POA), PA, OA, and SA from cells transfected with optoKiC and under 1 h illumination and darkness.

The images in Figure 1, Figure 2 and Figure 3 were analyzed with Fiji. The functions used for the quantitative analysis were Kymograph, Plot Profile, and Coloc2.

To calculate the changes in FA cluster distribution from the TOF-SIMS result, we involved Microsoft Excel (Microsoft, Redmond, WA, USA) and R (version 4.1.2) for the analysis.

To recognize the multi-FA clusters, we multiplied the signal intensities of two different lipids on the same pixel, the multiplication of which represents the clustering degree of those two lipids on that pixel. We used heat maps to better visualize the distribution of clusters.

To recognize the multi-FA clusters’ clustering degree, we performed the average nearest neighbor (ANN) analysis. Calculations were performed using the R package “spatstat” (Baddeley, A., Rubak, E., & Turner, R. (2015). Spatial Point Patterns: Methodology and Applications with R (Chapman & Hall/CRC Interdisciplinary Statistics) (1st ed.). Chapman and Hall/CRC.) and point plots were created to demonstrate the distribution. ANN ratio was calculated according to the equation [28].

For the LC-MS data, we applied LipidSearch™ software version 4.2.13 (Mitsui Knowledge Industry, Tokyo, Japan) [29] for lipid identification and quantification. We set the parameters as follows: database as higher-energy collisional dissociation (HCD); retention time as 0.01 min; search type as product_QEX; precursor tolerance as 5.0 ppm; and product tolerance as 8.0 ppm. Identification quality filters of A, B and C were applied. We used ±0.01 *m/z* tolerance and −1.0 min to 2.0 min of the retention time range for the quantification. After identification, alignment was performed with a 0.25 retention time tolerance.

After acquiring the annotated data from LipidSearch. We separated the data into different groups following the experimental conditions and made comparisons to seek the variations between these groups. To eliminate bias, both in and between the groups, we took advantage of normalization as the pre-treatment for the data. Normalization involved, in each group, dividing the intensities of each lipid by the total intensities of one class to which the lipid belongs. With the pre-treated data, we also used MS Excel to make volcano plots. We calculated the fold-change and *p* value of the *t*-test between different groups. Then, we used a scatter plot and set log_2_ fold-change as X and −log10 *p* value as Y to build the volcano plots.

## 3. Results

Our aim was to verify the FA cluster hypothesis through both observation and manipulation. FAs here refer to the FA chains contained in phospholipids rather than free FAs. As we already had a well-established method that enabled TOF-SIMS FA imaging [19], we first aimed to develop a tool that could change PM FA distribution by organelle translocation. Although the PM is mainly composed of lipids, the PM itself cannot synthesize vast quantities of lipids. Instead, its lipids are mainly acquired through lipid trafficking between intracellular organelles [30]. The ER, which is the center for de novo lipid synthesis, couples the synthesis and export of lipids [31,32]. We believed that the ER is the preferred organelle for manipulating the FA distribution through organelle translocation. Optogenetic tools with low invasiveness and high spatiotemporal precision are the optimal means to achieve this manipulation.

### 3.1. Design of an Optogenetic Tool to Manipulate FAs by Moving the ER

We designed an optogenetic tool called optoKiC. With illumination, we expected the optogenetic part to act as a bridge connecting the motor protein to the ER, and the motor protein to subsequently translocate the ER (Figure 1A). We made two plasmids to construct optoKiC: one containing a yellow fluorescent protein (Venus) at the N-terminal, with an improved light-inducible dimer (iLID) structural domain in the middle, whose affinity for SspB is altered more than 50-fold by blue light irradiation [33], and a fused cytochrome b5 (Cb5) at the C-terminal to anchor specifically to the ER membrane [34]. The other construct consisted of Kif1a at the N-terminal, a kinesin-3 family motor protein that moves continuously towards the positive end of the microtubule [25], a red fluorescent protein (RFP) in the middle, and SspB, a natural binding partner of the iLID at the C-terminal (Figure 1B). We imaged live cells introduced with the optoKiC. With 30.340 s of continuous blue-light irradiation, optoKiC changed the typical distribution of the ER (Figure 1C and Movie S1). For further comparison, we isolated and magnified a region (red frames) containing the center to the periphery and found that the ER was translocated to the cell periphery (Figure 1D). With the kymograph, we measured the sliding velocity of the bright spots on the ER during illumination, moving toward the cell periphery at an average speed of 0.64 µm/s. (Figure S1 left panel). The linear shape of the movement on the kymograph indicated that the movement was driven by a motor protein, probably Kif1a (Figure S1 right panel).

To quantitatively compare the ER distribution before and after irradiation, three regions of interest (ROIs) were taken in different orientations, and the fluorescence of ER was mapped (Figure 1E). The ER fluorescence signal in those ROIs showed peak shifts from the center of the cell to the periphery of 14.59, 5.44, and 2.22 µm, respectively, suggesting that optoKiC moves ER centrifugally (Figure 1F–H). We divided the cells into two parts by equal area: the perinuclear region and the pericellular region (Figure 1I). Under blue-light irradiation for 30.340 s, the ER fluorescence density in the perinuclear region of the left and the right cell decreased by 56.07 a.u./µm^2^ and 21.62 a.u./µm^2^, respectively. Simultaneously, the ER fluorescence density in the pericellular region increased by 31.68 a.u./µm^2^ and 20.93 a.u./µm^2^ (Figure 1J,K). These results indicated that a combination between Cb5 and Kif1a by optogenetic motifs could translocate the ER to the cell periphery within 30 s of blue-light illumination.

### 3.2. ER Translocation by optoKiC Is Reversible and Repeatable

The iLID-SspB dimer has been reported to be reversible and repeatable [33]. To find out whether optoKiC inherited the above properties of this dimer, we conducted blue light activation-darkness recovery-blue light activation on live cells and imaged the cells (Figure 2A). The ER moved to the cell periphery (T3–T6) under 108 s of blue-light irradiation, corresponding with the results presented in Figure 1. After the withdrawal of blue light (T7-T8), both fluorescence signals were restored to the state close to without manipulation. When optoKiC was activated again by blue light, the ER was translocated again to the pericellular region (T9-T10) (Figure 2B). To verify the interaction and dissociation of the two motifs, the colocalization of fluorescent signals representing the efficiency of protein interactions was quantified. We used Pearson’s *R* value to calculate the colocalization. The co-localization value increased with the presence of light and decreased with the termination of light, as shown in the chart, which is consistent with the fluorescence images (Figure 2C and Table S1). The above results indicated that the reversibility and repeatability of optoKiC were achieved using the light-dependent interaction of iLID-SspB.

### 3.3. Detection and Imaging of PM FA Clusters by TOF-SIMS

With the tool developed, we started the validation of the FA cluster hypothesis by imaging the PM FA distribution with and without manipulation. According to our previous report, we set up four FAs as our targets, namely: palmitoleic acid (POA), palmitic acid (PA), oleic acid (OA), and stearic acid (SA) [19]. We applied the developed optogenetic tools to the cells and manipulated FA clusters under two light conditions: 1-h continuous blue-light illumination (manipulated) and 1-h darkness (not manipulated).

Before examining the actual samples, we tested these three lipid standards in our possession and discovered that the standard PA, OA and SA, were detected by SIMS in their deprotonated form (Figure 3A–C). We also assayed the cell samples and observed signal peaks derived from POA, PA, OA and SA regardless of light conditions (Figure 3D–G).

**Figure 4:**
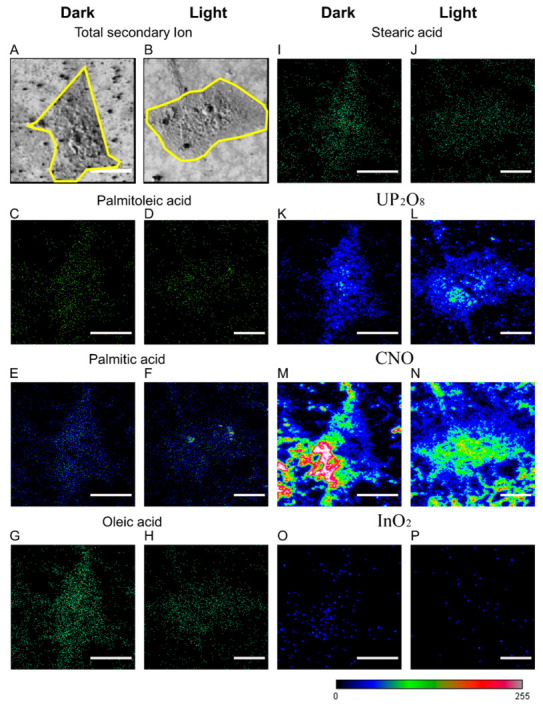
TOF-SIMS images of PM FAs. COS-7 cells were introduced with optoKiC under 1 h dark and light conditions and then imaged by TOF-SIMS. (**A**,**B**) The total secondary ion images of the cells under dark and light conditions (Yellow frames: Cell outlines). (**C**–**J**) The ion images of POA, PA, OA, and SA under dark and light conditions. (**K**–**P**) The ion images of UP_2_O_8_ (signal from uranium salt for fatty acid fixation), CNO (signal from biomolecules, such as protein), and InO_2_ (signal from ITO glass and background noise) under dark and light conditions. Scale bars: 25 µm.

Based on the successful detection of PM FAs, we imaged the cells by TOF-SIMS. The images of the total secondary ions, which originated from all regions of the sample presented and reflected the cell profile (Figure 4A–B). Different FA species displayed diverse distribution patterns in the images of the four FAs, each of which exhibited a similar outline to the cell (Figure 4C–J). To verify the accuracy of the imaging, we visualized signals from non-FA sources. The signals originating from biological molecules (Figure 4K–N) all showed the same profile as the cells under dark and light conditions, respectively (Figure 4A–B). The signals originating from glass or background noise showed a completely irregular distribution (Figure 4O,P). The images of the three non-FA signals served as control groups to further demonstrate the accuracy of TOF-SIMS detection and imaging.

Thus far, we have imaged the PM FAs by TOF-SIMS with or without optical manipulation. From the images, we discovered that all four FAs appeared to be distributed as clusters and showed an altered pattern with optical manipulation.

### 3.4. Optical Manipulation Changed PM FA Cluster Distribution

**Figure 5:**
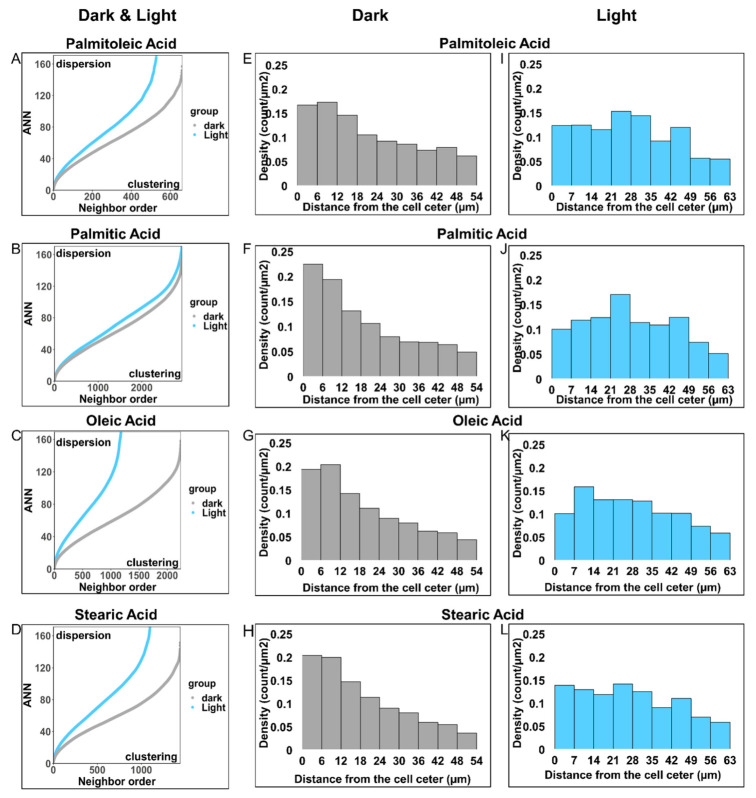
Quantitative judgment description, and comparison of PM FA cluster distribution. (**A**–**D**) The average nearest neighbor (ANN) vs. neighbor order plots (ANN curves) of POA, PA, OA, and SA under dark and light conditions prove that they existed as clusters. (**E**–**H**) The density histogram shows the distribution of POA, PA, OA, and SA clusters under dark conditions. (**I**–**L**) The density histogram shows the distribution of POA, PA, OA, and SA clusters under light conditions. Scale bars: 25 µm.

To quantitatively describe and judge the distribution pattern of PM FAs, we performed an average nearest neighbor (ANN) analysis on the PM FAs. ANN analysis revealed whether the molecules were clustered on a plane and the degree to which the molecules were clustered. The ANN ratios of all four FAs were less than 1 in both dark and light conditions (Table S2). The ANN ratio of less than 1 means the molecules were clustered. Thus, the above results proved that all four PM FAs exist as clusters under both conditions. Furthermore, the ANN curves of all four FAs under light manipulation appeared above the ANN curves of the four FAs without light (Figure 5A–D). These results indicated that the optical manipulation made four PM FA clusters more dispersed. All results above were reproducible (Table S3 and Figure S2).

We further investigated how optoKiC changed the distribution pattern of PM FA clusters, resulting in a lower clustering degree. For this purpose, we divided a cell into nine ROIs equally spaced from the center to the periphery. We then calculated the density by summing the FA count (intensity) in each ROI and dividing them by the area of the ROI. After obtaining the ROI’s density, we normalized it by the sum of all ROI densities. (Figure S3). Finally, we used the normalized density to describe the distribution of PM FA clusters.

We applied histograms to depict the distribution of FA clusters on the PM. In dark conditions, the density of all FA clusters was distributed in descending order from the cell center to the cell periphery, and different FAs showed distinct distribution patterns (Figure 5E–H). Under illumination, the highest density region of POA clusters, PA clusters, OA clusters, and SA clusters shifted from the 0–12 μm interval, 0–6 μm interval, 0–12 μm interval, and 0–12 μm interval to 21–35 μm interval, 21–28 μm interval, 7–28 μm interval, and 21–35 μm interval, respectively (Figure 5E–L). All the FA clusters’ distribution pattern changes were reproducible (Figure S4A–H). We also calculated and compared the biomolecule-derived signal distributions, such as protein (CNO) and uranium salts bound to the phosphate group of phospholipids (UP_2_O_8_), respectively. The CNO signals demonstrated the highest density region at the 12–18 μm interval, which is reasonable since the protein synthesis site, ER, is located near the cell center (Figure S5A). The signal of UP_2_O_8_ showed a similar pattern to the four FAs, as it bound to phospholipids (Figure S5B). After light irradiation, CNO showed a relatively uniform distribution due to the translocation of the ER (Figure S5A,C). The signal from uranium salt showed a similar distribution change to that of FAs under illumination (Figure S5B,D). All the non-FA signal distribution pattern changes were reproducible (Figure S6A–D). These non-FA results supported the accuracy of the imaging and calculation method.

The preceding result indicated that PM FAs exist as clusters and are also manipulated as clusters. Furthermore, the distributions of PM FA clusters were altered by optoKiC into a relatively homogeneous pattern upon 1 h illumination.

### 3.5. optoKiC Changed Multi-FA Clusters Distribution on PM

**Figure 6:**
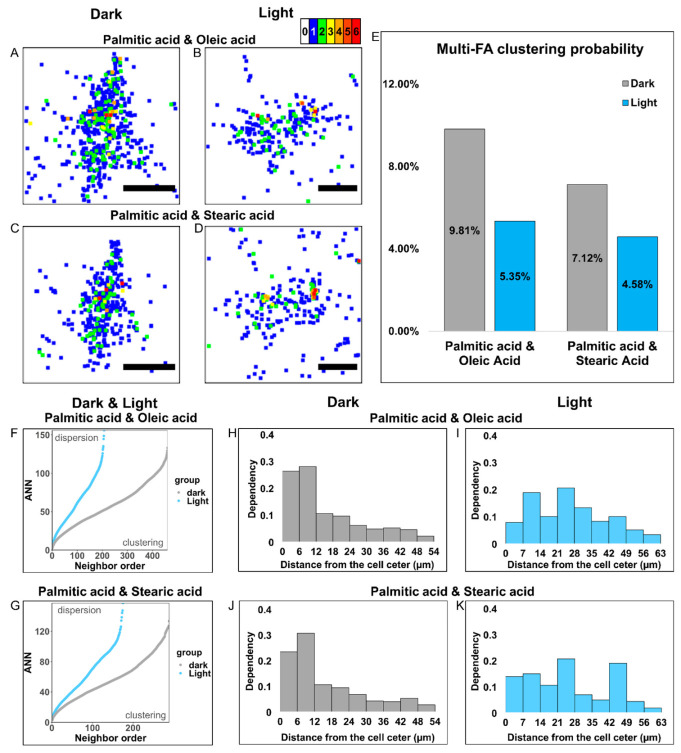
Imaging and manipulation of multi-FA clusters. (**A**,**B**) The heat map imaging of the multi-FA clusters formed by PA and OA. (**C**,**D**) The heat map imaging the multi-FA clusters formed by PA and SA. (**E**) The bar graph shows the probabilities to form multi-FA clusters by PA and OA/SA under dark and light conditions. (**F**–**G**) The ANN curves of multi-FA clusters formed by PA and OA/SA under dark and light conditions proves that they exists as clusters. (**H**–**I**) The density histograms show the distribution of PA-OA-formed multi-FA clusters under both dark and light conditions. (**J**–**K**) The density histograms show the distribution of PA-SA-formed multi-FA clusters under both dark and light conditions. Scale bars: 25 µm.

It is well known that phospholipids usually contain two FA chains. Therefore, we investigated the distribution pattern of the co-localized FAs on the PM. We recognized two FAs appeared on the same pixel as a multi-FA cluster. The multiplication of the two FA *m/z* values was used as the intensity of the multi-FA cluster (Figure S7). We imaged the multi-FA clusters using heat maps and observed that the heat maps exhibited more multi-FA clusters under no light conditions (Figure 6A–D). We calculated the formation probability of multi-FA clusters. The probability of PA and OA forming multi-FA clusters reduced by 4.46% under optical manipulation compared to dark conditions. While the probability of PA and OA forming multi-FA clusters reduced by 2.54% with optical manipulation (Figure 6E). The formation probability of multi-FA clusters reduced reproducibly under blue-light illumination (Figure S8).

Likewise, we employed the same analytical technique used for FA clusters to describe and compare the distribution of multi-FA clusters under different conditions. With the ANN analysis, the ANN ratio of both multi-FA clusters was less than 1, which proved that they existed as clusters on the PM (Table S4). Interestingly, the ANN curves showed that the multi-FA clusters became more dispersed under optical manipulation, similar to the FA clusters (Figure 6F,G). The ANN analysis results were reproducible (Table S5 and Figure S9). We then investigated the multi-FA distribution pattern by density histogram. Under dark conditions, both multi-FA clusters were more concentrated in the center of the cell at 0–12 μm interval, while with illumination, the high-density region shifted to the 21–28 μm interval (Figure 6H–K). The distribution pattern changes of the multi-FA clusters under optical manipulation demonstrated reproducibility (Figure S10). The above findings indicated that the PM FAs could co-localize and form multi-FA clusters. Furthermore, the multi-FA cluster distribution patterns were altered in a relatively homogeneous way by optical manipulation.

### 3.6. The Manipulation of PM FAs Barely Changed the Cellular Lipid Composition

**Figure 7:**
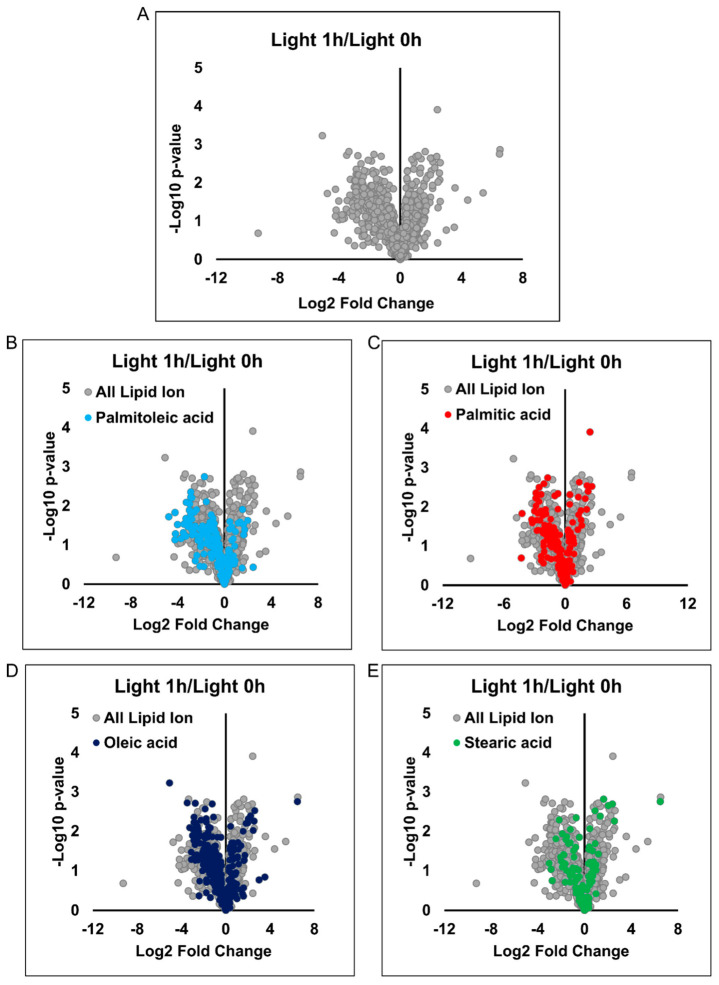
LC-MS/MS showing the cellular lipid composition barely changed due to optoKiC. (**A**) Volcano plot between Light 1-h (illuminated for 1 h) and Light 0-h (keep in dark for 1 h). (**B**) Volcano plot between Light 1-h and Light 0-h in which lipids containing 16:0 palmitic acid are labeled in red. (**C**) Volcano plot between Light 1-h and Light 0-h in which lipids containing 16:1 palmitoleic acid are labeled in light blue. (**D**) Volcano plot between Light 1-h and Light 0-h in which lipids containing 18:1 oleic acid are labeled in dark blue. (**E**), Volcano plot between Light 1-h and Light 0-h in which lipids containing 18:0 stearic acid are labeled in green.

Now that we have manipulated the distribution of both FA clusters and multi-FA clusters by optoKiC, the next question is whether significant differences in the lipid composition of the cells occurred when the distribution changed. For this purpose, we extracted whole lipids from cells by the Bligh–Dyer method and performed LC-MS/MS to determine the lipids [27]. Afterwards, we applied annotation of lipid species using LipidSearch software [29,35]. Finally, we visualized the comparison between the illuminated (manipulated) and non-illuminated (not-manipulated) groups through volcano plots. Same as when changing the distribution, we kept the illumination time at 1 h. As shown in the figure, most of the scatters were closely distributed on both sides along the *Y*-axis (Figure 7A). We calculated and ranked all lipids proportionally according to their fold-change, with 99.29% having a 16-fold change and 91.45% not having a 4-fold change. Even when we reduced the fold-change to 2-fold, 79.35% of the lipids in the cells still did not have this change (Table S6).

We manipulated four FAs, thus further investigating the compositional changes of lipids containing these four FA chains. We labeled the lipids containing these FA chains in the volcano plot and listed their percentages. Lipids containing palmitoleic, palmitic, oleic, and stearic acids accounted for 15.87%, 19.94%, 32.15%, and 13.12% of the total number of lipid species, respectively (Table S7). After labeling these four chains, lipids carrying distinct FAs stayed on both sides close to the *Y*-axis of the volcano plot (Figure 7B–E).

The above results clarified that when the distribution of PM FAs was altered by optoKiC, the composition of most lipids changed less than 2-fold. This result supports that the changes in the distribution of FA clusters are changes in distribution rather than a projection of cytoplasmic component changes on the cell surface.

## 4. Discussion

In this study, we revealed the PM heterogeneity from the FA perspective. From the homogeneous “fluid mosaic” model to the “lipid raft” model to the present, the study of membrane heterogeneity has drawn numerous interests [3,7,36]. With the development of technology, an increasing number of techniques have been applied to the observation of rafts/microdomains/PM heterogeneity [37]. We found that FAs in the PM existed as clusters that were heterogeneous. The PM FA clusters showed a cell center-concentrated distribution which was consistent with the previous report that glycosylphosphatidylinositol (GPI)-anchored protein (a representative of lipid rafts) preferred to be distributed around the cell center [8]. We believe that FA clusters may be a reflection of lipid rafts from the rafts’ FA perspective.

Attempts to manipulate PM heterogeneity have never ceased, and experiments to manipulate PM heterogeneity by altering the cholesterol/ganglioside/FA composition of the PM are gradually elucidating the causes of PM heterogeneity [38]. Our study reported the manipulation of PM heterogeneity in the form of FA clusters. Under optical manipulation, the PM FA clusters were distributed in a more dispersed way. We found that the FA clusters were distributed in a relatively homogeneous pattern on the PM when the FA showed a more dispersed state under light manipulation. These results suggested that the specific FA clusters’ distribution could be a potential cause of PM heterogeneity formation. Overall, we offer a novel opportunity to study membranes and membrane lipids, the distribution of FAs, and furnish a potent tool for their investigation.

Our multi-FA clusters model also helps to explain the PM heterogeneity and supports the lipid raft hypothesis. Lipid raft theory believes that saturated FA clustering promotes the formation of lipid rafts/membrane domains by pulling the lipid molecules together, while monounsaturated FAs contribute to the formation of lipid rafts by pushing the raft molecules [39,40,41,42,43]. The PA of saturated FAs is essential for protein palmitoylation, which regulates the raft affinity of most integrated raft proteins [44]. It can be considered that PA is one of the vital functional molecules on lipid rafts. SA is also a common saturated FA in the body. PA and SA can co-localize and thus form multi-FA clusters. Clusters formed between saturated FAs have a tighter lipid packing and may provide the physical basis for lipid raft/membrane domain formation (pulling). Interestingly, PA and OA, which are saturated and monounsaturated FAs, can also form multi-FA clusters. We suggest that these multi-FA clusters could represent non-raft regions (lipid oceans) and help raft formation (pushing). In the absence of manipulation, PA and OA are more likely to form multi-FA clusters than PA and SA. This result is consistent with the speculation that the proportion of “lipid oceans” is higher than that of “lipid rafts” in lipid raft theory. In other words, the pushing molecules should surround the molecules to be pushed.

Furthermore, in terms of lipidomics for both multi-FA clusters, PA is the most common saturated FA among the FAs in the body [45]. In addition, phosphatidylcholine, which accounts for over 50% of the membrane’s composition, mostly has a cis-unsaturated FA acyl chain [30]. Studies have shown that PA, OA, and SA are all dominant in the PM or membrane domain [46]. The abundance enrichment provides a rational reason for the formation of clusters of these three FAs. In addition, a mechanism exists for cells to regulate the content of PA, the metabolite of which is OA and SA [47,48]. This may be one reason for their physical proximity and thus co-localization into multi-FA clusters. In the FA composition of the PM or membrane domain, OA has a higher ratio compared to SA. This is one of the reasons why PA and OA have a higher probability of forming clusters.

Under light manipulation, the formation probability of both multi-FA clusters was reduced to a similar level. Comparisons of the distribution patterns of multi-FA clusters revealed that their heterogeneity had changed, as had their clustering degree. These results suggest that one way for cells to regulate PM heterogeneity via FAs is to prevent them from forming both FA clusters and multi-FA clusters. Thus, they cannot form a specific phase or physical state, much less constitute a specific microdomain (raft) that can be maintained [49]. Excess lipid rafts play a vital role in numerous diseases and our tool may offer potential therapeutic approaches for diseases such as inflammation, Alzheimer’s disease, and others [50,51,52].

The limitations of the ionization capacity led to the imaging of only four species of FAs in this study. With further development of mass spectrometry, we can image more FA (e.g., linoleic acid and EPA) on PM and provide a more comprehensive description of PM lateral heterogeneity from an FA perspective in the near future. In addition, we are very much looking forward to validating and comparing our interpretation of membrane heterogeneity from a FA perspective with studies from a lipid raft/lipid microdomain perspective. For example, in the imaging of cholesterol and GM1 colocalization on the membrane by Nano-SIMS, the localized region can be considered as a manifestation of rafts [53]. In addition, TOF-SIMS, as a surface imaging technique, is currently difficult to accurately measure the exact depth of the measurement. We have considered using both TOF-SIMS and GCIB-TOF-SIMS to image the upper part of the cell at multiple levels in future studies to obtain a more comprehensive understanding of the cell membrane.

Our manipulation of PM FA distribution was achieved by translocating the ER to the cell periphery. The mechanism behind this manipulation is believed to be interesting and complex.

The movement of the ER towards the cell periphery would cause the physical proximity of the ER to the PM. The membrane contact site (MCS) is a region where the two membranes are extremely close to one another, usually less than 30 nm [54]. Our translocation of the ER may promote the formation of the PM-ER MCS. The increase in the MCS would affect lipid transport and thus alter the distribution of FAs in the PM [55,56]. However, the MCS is also responsible for cholesterol transport [57]. Cholesterol enhances the clustering effect of lipids through the umbrella effect [58]. In our results, the possibility of forming multi-FA clusters on the PM and the clustering degree of FAs both decreased after optical manipulation. These results imply that the level of cholesterol in the PM is not increased, which is in contrast to the MCS formation. Thus, the changes in MCS need to be verified by further experiments.

From the perspective of lipid rafts/microdomains, the ER also has “raft”-like microdomains, and the ER-rafts may highly associate with PM-rafts [59,60]. By moving the ER to the cell periphery, we also move the rafts on the ER towards the edge at the same time. The change in the distribution of ER-rafts may further transform into the distribution change of PM-rafts, ultimately changing the distribution of PM FAs through the rafts.

Phase separation is the basis of various membrane behaviors, and phase separation is also an important cause of lipid raft formation. Our results revealed the distribution of PM FAs, and that heterogeneous distribution may be the physical basis of phase separation. In addition, our tool for ER translocation offers the possibility of studying the role of MCS in phase separation.

## 5. Conclusions

We revealed the PM FAs exist as FA clusters and manipulated the PM FA cluster distribution by light manipulation using optoKiC. We also found FAs co-localize and form multi-FA clusters. The distribution of PM multi-FA clusters was altered by optical manipulation.

## Data Availability

All data needed to evaluate the conclusions in the paper are present in the paper and/or the Supplementary Materials. Additional data related to this paper may be requested from the authors.

## Supplementary Materials

The following supporting information can be downloaded at: https://www.mdpi.com/article/10.3390/cells12010010/s1, Figure S1: Kymograph testing motility of bright spots on ER during the illumination.; Figure S2: Reproducibility of ANN analysis of FA clusters.; Figure S3: The schematic of calculation and description for density based distribution pattern comparison using data acquired from TOF-SIMS.; Figure S4: TOF-SIMS images of PM FAs. Reproducible changes of FA clusters distribution pattern under optical manipulation.; Figure S5: Non FA signals distribution comparison.; Figure S6: Reproducibility of non FA signals distribution alteration with optical manipulation.; Figure S7: The schematic of calculation and description for multi-FA clusters distribution using data acquired from TOF-SIMS.; Figure S8: Reproducibility of the bar graphs showing the probabilities to form multi-FA clusters by PA and OA/SA under dark and light conditions.; Figure S9: Reproducibility of ANN analysis of multi-FA clusters.; Figure S10: Reproducibility of the distribution pattern change of the multi-FA clusters. Table S1: The Pearson’s R-value of two fluorescence from Venus-iLID-Cb5 and Kif1a-RFP-SspB.; Table S2: The ANN ratio proving FAs on PM showed clustered status.; Table S3: The reproducibility data of FAs ANN ratios indicated the FAs clustered.; Table S4: The ANN ratio proving multi-FA clusters existing as clusters on PM.; Table S5: The ANN ratio proving multi-FA clusters existing as clusters on PM reproducibly.; Table S6: The lipid composition changes of cellular lipids.; Table S7: The lipid composition for lipids containing different FAs.; Video S1: Movies showing the ER translocation with blue light illumination.
