# Supplementary material for: Imaging and Manipulation of Plasma Membrane Fatty Acid Clusters Using TOF-SIMS Combined Optogenetics
Source: Cells. 2022 Dec 20;12(1):10. doi: 10.3390/cells12010010 (PMC9818728; doi:10.3390/cells12010010)
Supplement: Supplementary file 1 [file cells-12-00010-s001.zip › 221017_Supplementary_Materials_ZC.pdf]

cells

MDPI

Supplementary Materials for the manuscript:

Imaging and manipulation of plasma membrane fatty acids clusters using TOF-SIMS combined optogenetics

The supplementary materials contains:

Supplementary figures: S1-S10

Supplementary Tables: Table S1-S7

Supplementary Movies: Movie S1

Supplementary Figures of the manuscript

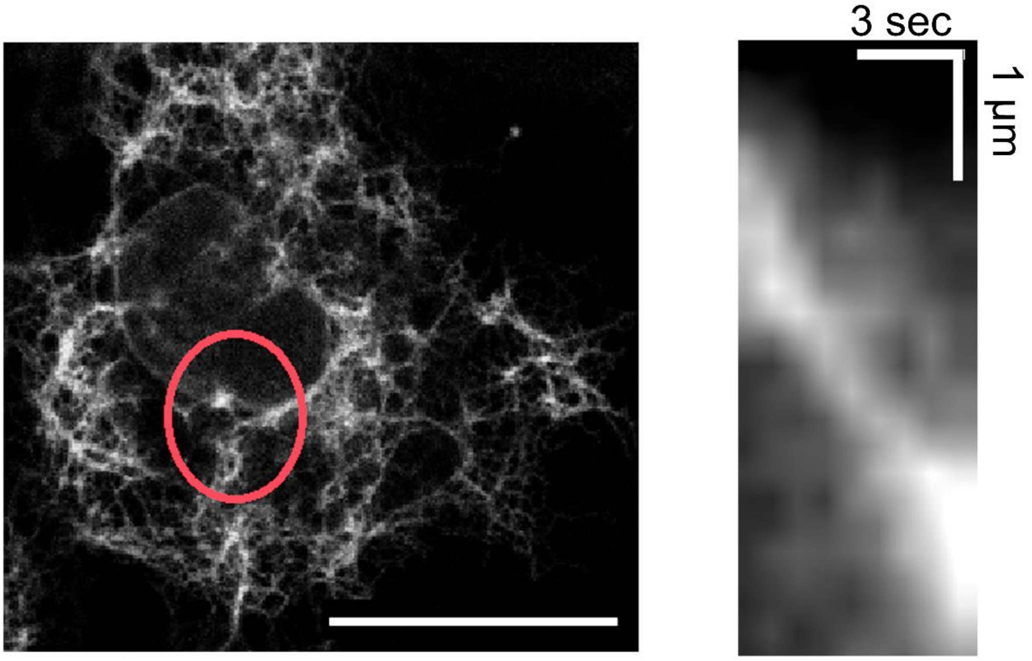

Supplementary Figure S1. Kymograph testing motility of bright spots on ER during the illumination. Kymograph applied to quantitatively test the motility of the ER translocation (Red circle: a bright spot on ER). Scale bar in the left panel: 25 μm, horizontal bar in the right panel: 3-sec, vertical bar in the right panel: 1 μm.

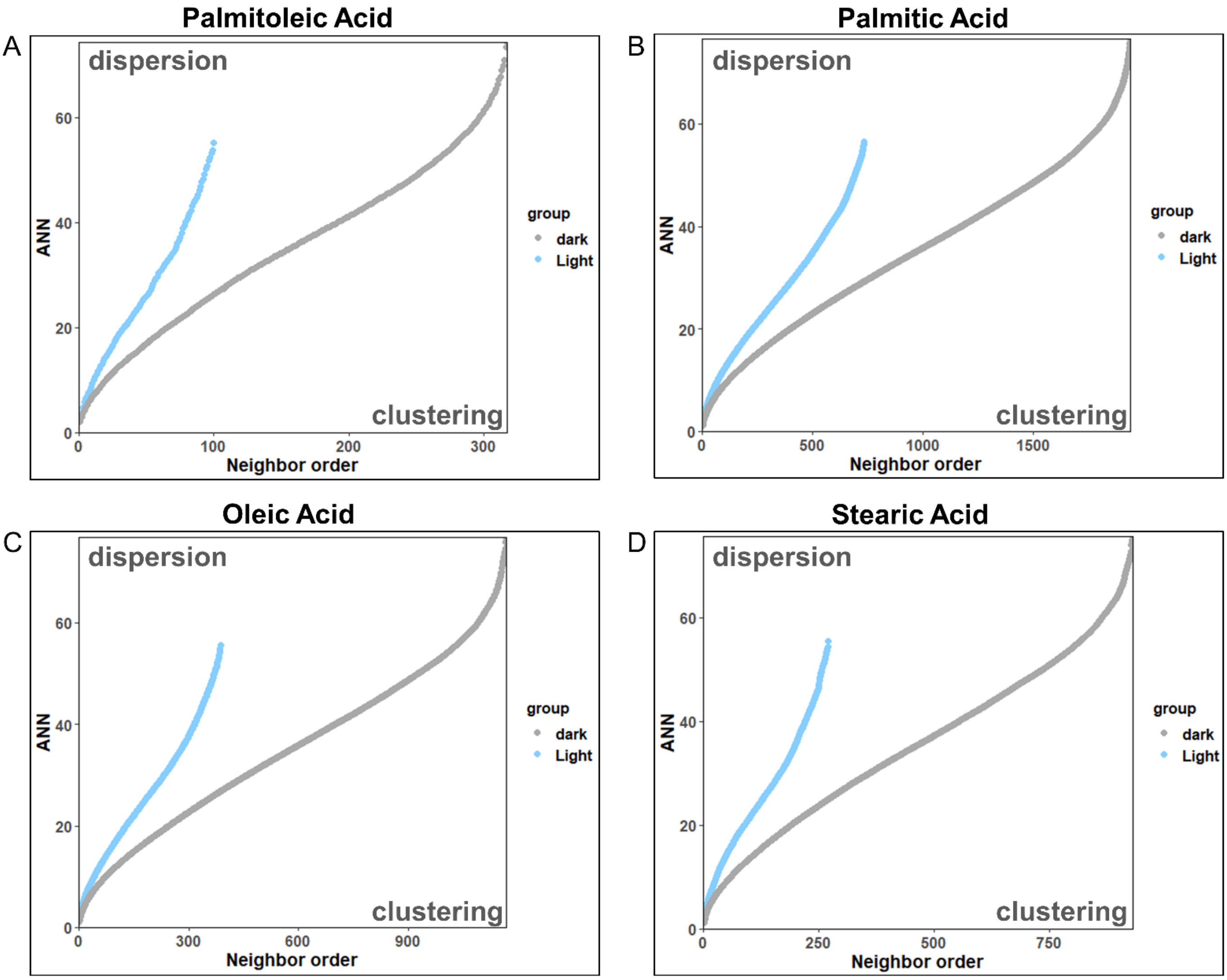

Supplementary Figure S2. Reproducibility of ANN analysis of FA clusters. (**A-D**) The average nearest neighbor (ANN) vs neighbor order plots (ANN curves) of POA, PA, OA, and SA under dark and light conditions.

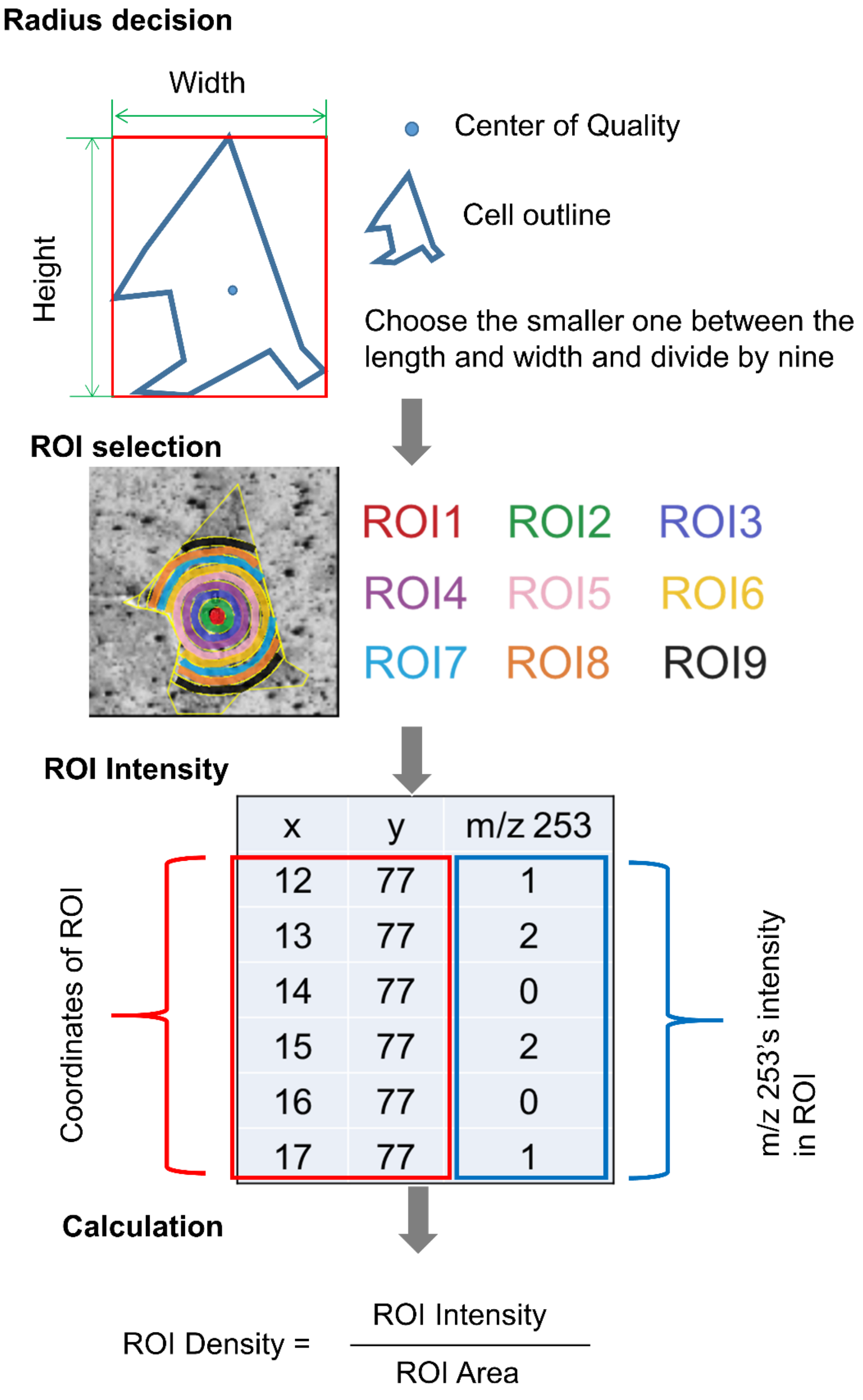

Supplementary Figure S3. The schematic of calculation and description for density based distribution pattern comparison using data acquired from TOF-SIMS. We measured the width and height of the cell and choose the smaller one for radius calculation. We drew ROIs using the center of quality as the center of the ROIs. Use width/height to divided by nine as the radius and interval of radius between two ROIs. We separated the cell from the center to the peripheral by nine concentric circles with equal radius differences. The ROI number corresponding to the area through different colors. We then sum the intensity of each ROI and divided it by the ROI's area. After that we normalized the density by their sum.

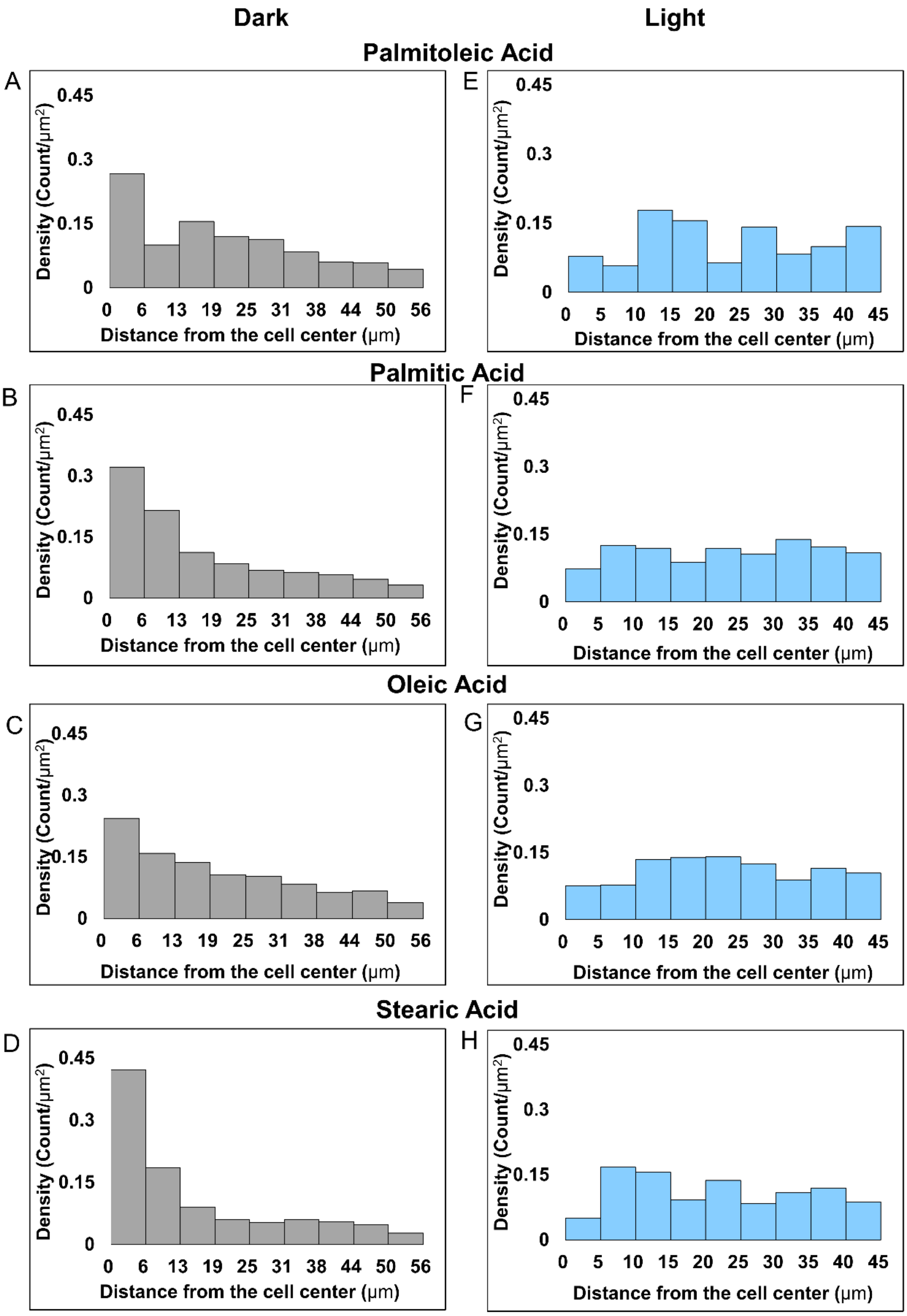

Supplementary Figure S4. TOF-SIMS images of PM FAs. Reproducible changes of FA clusters distribution pattern under optical manipulation. (**A-D**) The density histogram showing the distribution of POA, PA, OA, and SA clusters under dark conditions. (**E-H**) The density histogram showing the distribution of POA, PA, OA, and SA clusters under light conditions.

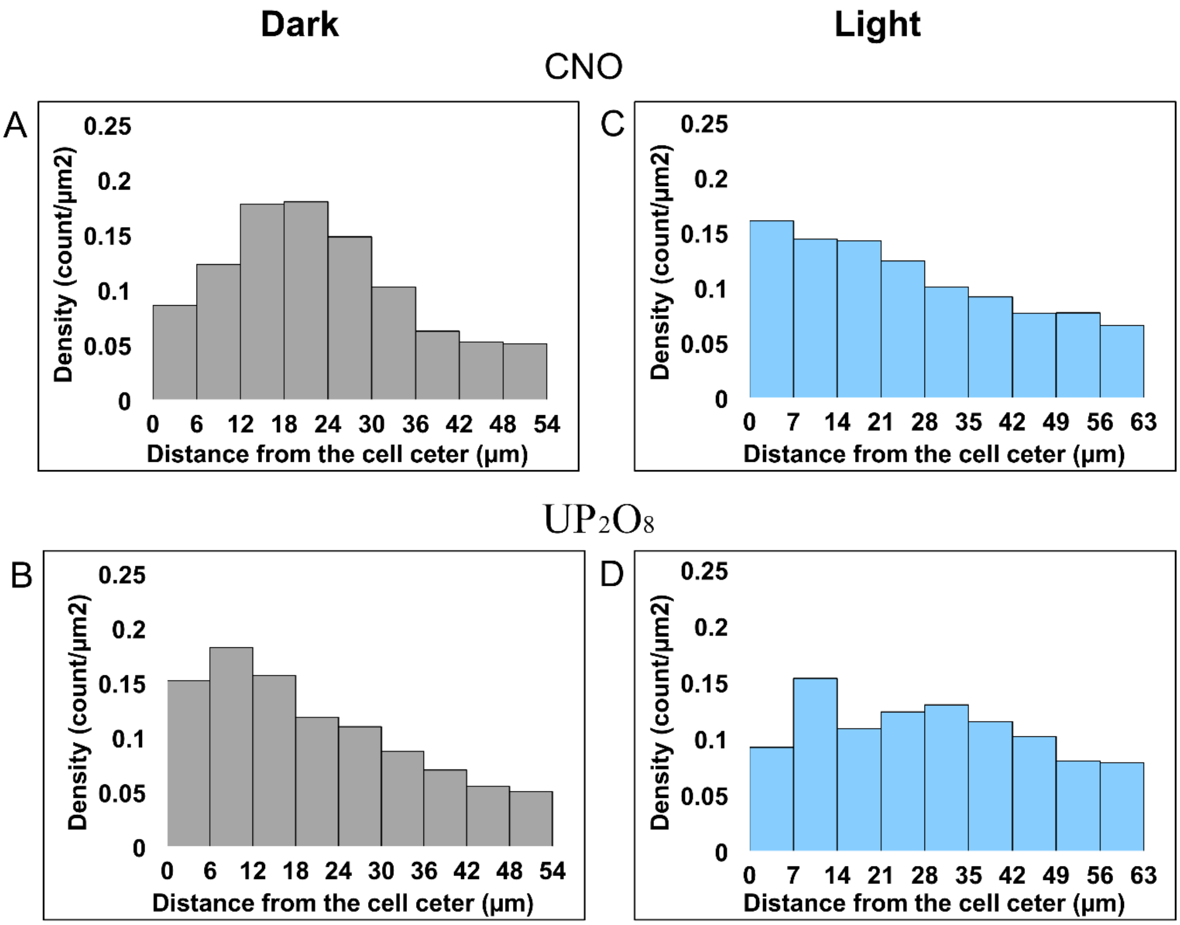

Supplementary Figure S5. Non FA signals distribution comparison. (**A-B**) The density histogram showing the distribution of CNO (biomolecule derived) and UP_2O_8 (uranium salt for fatty acids fixation) under dark conditions. (**C-D**) The density histogram showing the distribution of CNO (biomolecule derived) and UP_2O_8 (uranium salt) under light conditions.

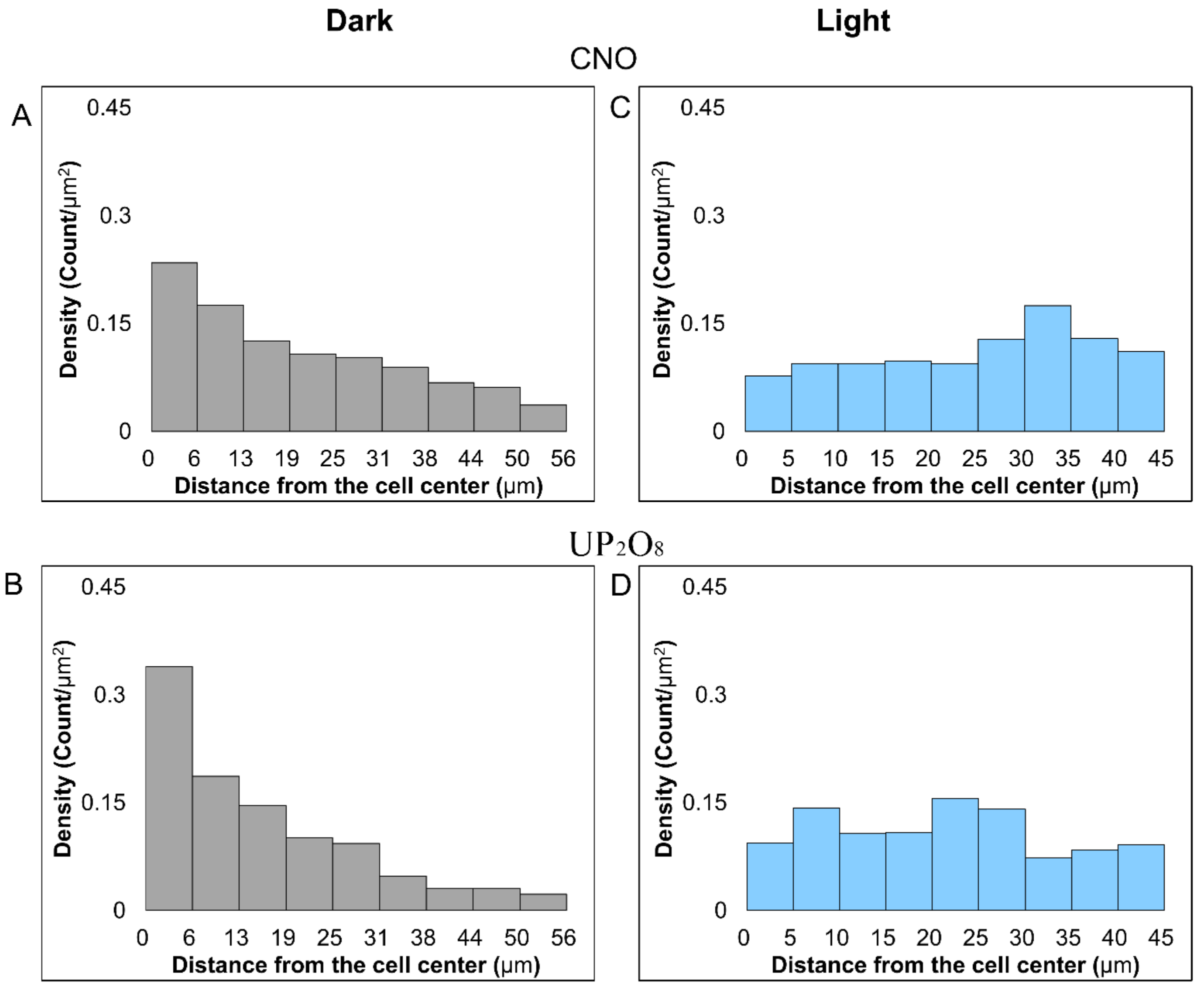

Supplementary Figure S6. Reproducibility of non FA signals distribution alteration with optical manipulation. (**A-B**) The density histogram showing the distribution of CNO (biomolecule derived) and UP_2O_8 (uranium salt for fatty acids fixation) under dark conditions. (**C-D**) The density histogram showing the distribution of CNO (biomolecule derived) and UP_2O_8 (uranium salt) under light conditions.

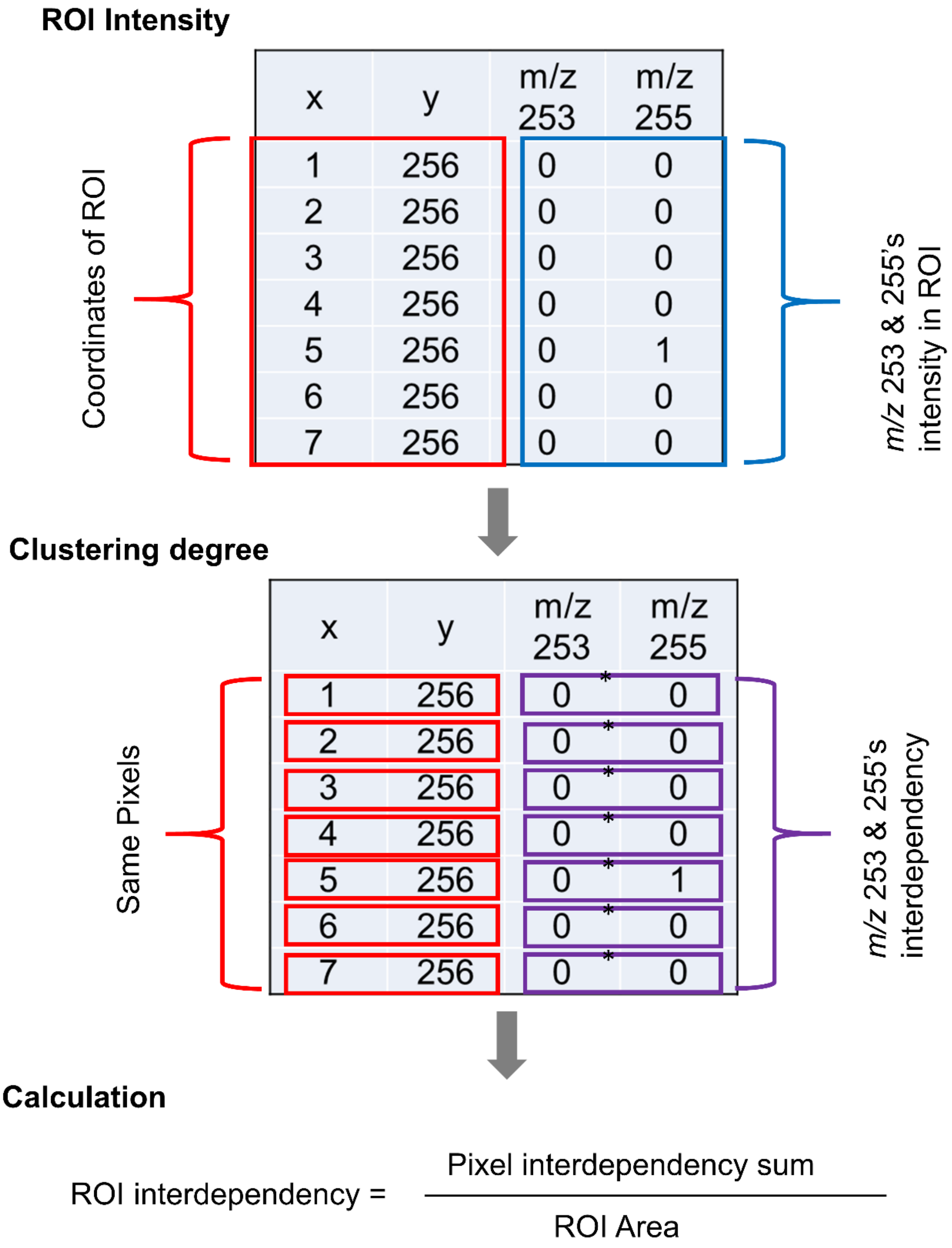

Supplementary Figure S7. The schematic of calculation and description for multi-FA clusters distribution using data acquired from TOF-SIMS. We multiplied the m/z values belongs to the same XY coordinate which means in same pixel. The multiplied result indicated the intensity of the multi-FA clusters. We then sum the multi-FA's intensity in the ROIs and divided them by the ROI's area for normalization. Similarly, the distribution of multi-FA clusters was described as density.

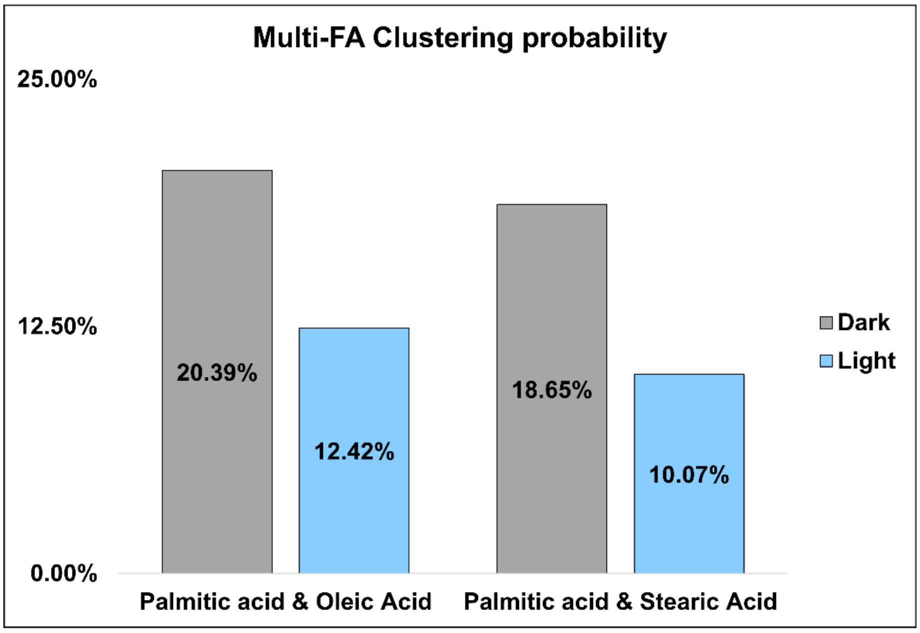

Supplementary Figure S8. Reproducibility of the bar graphs showing the probabilities to form multi-FA clusters by PA and OA/SA under dark and light conditions.

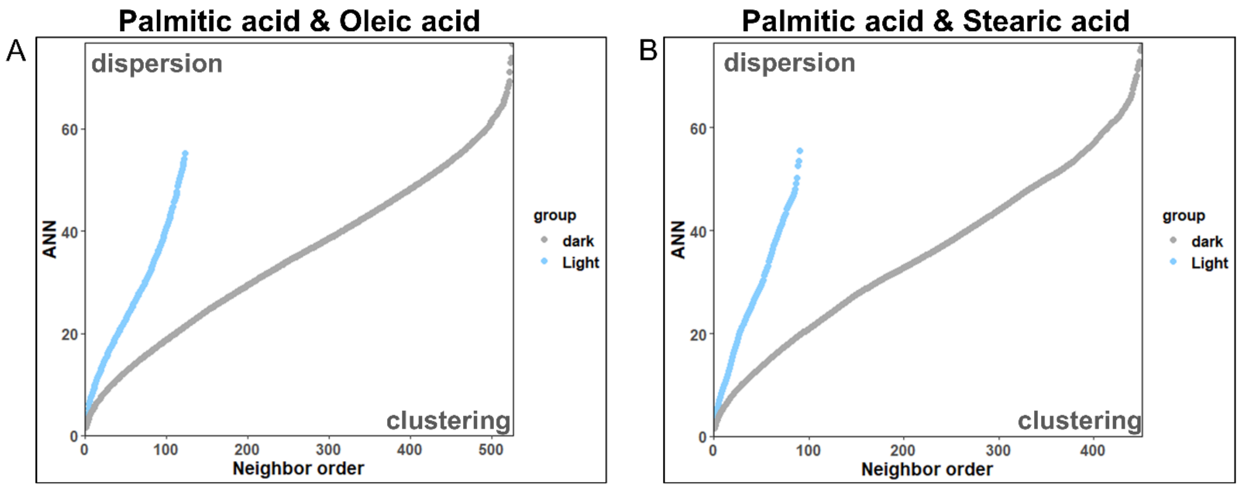

Supplementary Figure S9. Reproducibility of ANN analysis of multi-FA clusters. (**A-B**) The ANN vs neighbor order plots of multi-FA clusters formed by PA and OA/SA under dark and light conditions.

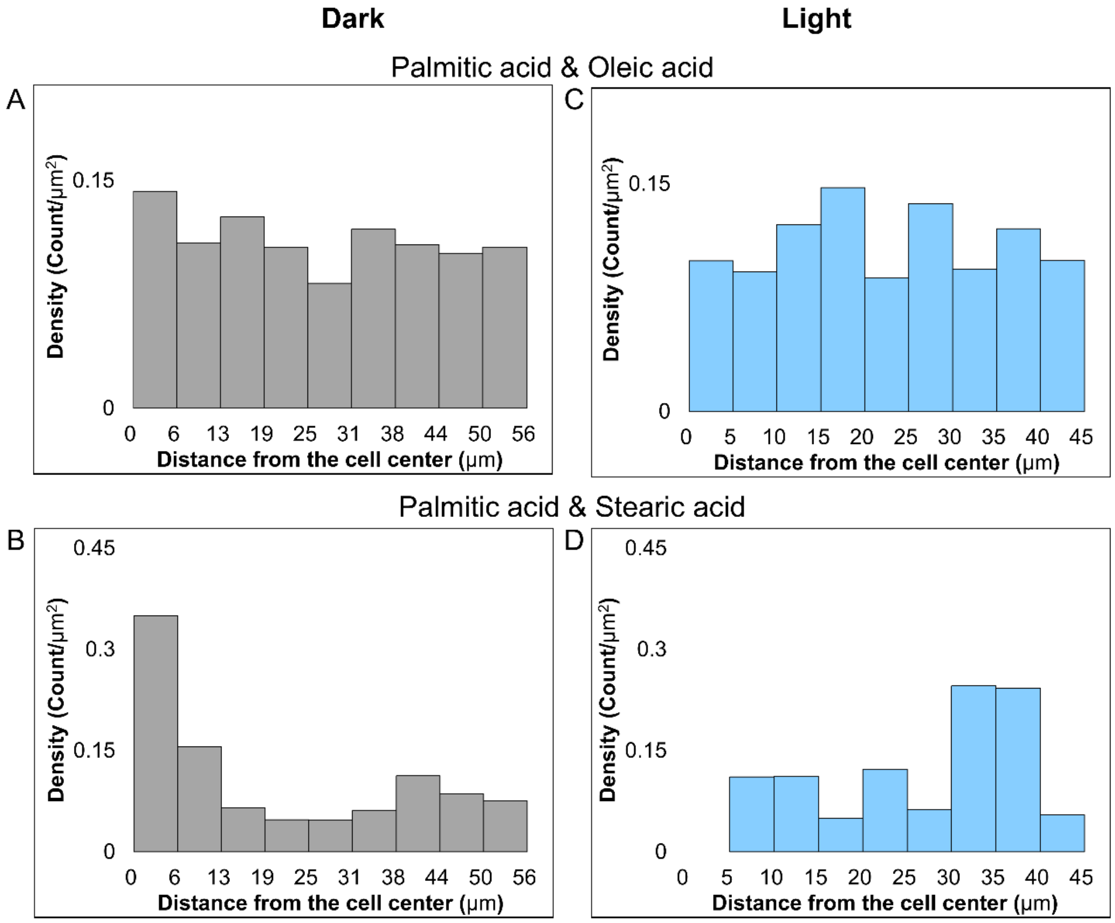

Supplementary Figure S10. Reproducibility of the distribution pattern change of the multi-FA clusters. (A-B) The density histogram of multi-FA clusters formed by PA and OA/SA under dark condition. (**C-D**) The density histogram of multi-FA clusters formed by PA and OA/SA under light condition.

R-Value	Cell 1	Cell 2	Cell 3	Cell 4	Cell 5	Cell 6	Cell 7
**T3**	0.61	0.77	0.66	0.78	0.78	0.79	0.65
**T6**	0.67	0.85	0.73	0.83	0.82	0.82	0.69
**T9**	0.63	0.79	0.66	0.78	0.72	0.76	0.62
**T10**	0.70	0.83	0.78	0.82	0.72	0.78	0.74

Table S1. The Pearson's *R*-value of two fluorescence from Venus-iLID-Cb5 and Kif1a-RFP-SspB. The colocalization of the two fluorescence was represented by correlation value.

Fatty Acid	Dark	Light
**Palmitoleic Acid**	0.00206	0.00233
**Palmitic Acid**	0.00229	0.00252
**Oleic Acid**	0.00217	0.00232
**Stearic Acid**	0.00209	0.00240

Table S2. The ANN ratio proving FAs on PM showed clustered status. The ANN ratio of FAs under dark and light conditions smaller than 1.

ANN ratio	Dark	Light
**Palmitoleic Acid**	0.0104	0.0164
**Palmitic Acid**	0.0122	0.0175
**Oleic Acid**	0.0104	0.0162
**Stearic Acid**	0.0104	0.0155

Table S3. The reproducibility data of FAs ANN ratios indicated the FAs clustered. The ANN ratio of FAs under dark and light conditions smaller than 1.

Lipid Clusters	Dark	Light
**Palmitic Acid & Oleic Acid**	0.00175	0.00193
**Palmitic Acid & Stearic Acid**	0.00176	0.00194

Table S4. The ANN ratio proving multi-FA clusters existing as clusters on PM. The ANN ratio of PA & OA clusters and PA & SA clusters under dark and light conditions smaller than 1.

ANN ratio	Dark	Light
**Palmitic Acid & Oleic Acid**	0.0095	0.0160
**Palmitic Acid & Stearic Acid**	0.0093	0.0151

Table S5. The ANN ratio proving multi-FA clusters existing as clusters on PM reproducibly. The ANN ratio of PA & OA clusters and PA & SA clusters under dark and light conditions smaller than 1.

Category	Count	Percentage
Total	983	100.00%
Fold change<2	780	79.35%
Fold change<4	899	91.45%
Fold change<16	976	99.29%

Table S6. The lipid composition changes of cellular lipids. Almost 80 percent of lipids did not show a composition change more than twice.

Species (FA contained)	Percentage in all lipids	Fold change < 2	Fold change < 4	Fold change < 16
**Palmitoleic acid**	15.87%	73.08%	85.26%	98.72%
**Palmitic acid**	19.94%	73.98%	85.71%	99.49%
**Oleic acid**	32.15%	73.10%	87.97%	99.37%
**Stearic acid**	13.12%	76.74%	94.57%	99.22%

Table S7. The lipid composition for lipids containing different FAs. The proportion of lipids containing the four FAs showed little of change either.

Movie S1.

Movies showing the ER translocation with blue light illumination. Channel of Cb5, Kif1a and Merge. Scale bars: 25μm.
